# Tactile Sensor-Based Body Center of Pressure Estimation System Using Supervised Deep Learning Models

**DOI:** 10.3390/s26010286

**Published:** 2026-01-02

**Authors:** Jaehyeon Baik, Yunho Choi, Kyung-Joong Kim, Young Jin Park, Hosu Lee

**Affiliations:** 1 Department of Control and Robot Engineering, Gyeongsang National University, Jinju 52828, Republic of Korea; bjhme22@naver.com; 2 Department of Electrical and Computer Engineering, University of Washington, Seattle, WA 98195, USA; dbsh070@gmail.com; 3 Department of AI Convergence, College of Information and Computing, Gwangju Institute of Science and Technology, Gwangju 61005, Republic of Korea; kjkim@gist.ac.kr; 4 School of Mechanical Engineering, Gyeongsang National University, Jinju 52828, Republic of Korea; youngjin.park@gnu.ac.kr; 5 Gyeongnam Aerospace & Defence Institute of Science and Technology, Gyeongsang National University, Jinju 52828, Republic of Korea

**Keywords:** balance, center of pressure, estimation, tactile sensor, supervised learning, ResNet-Bi-LSTM, CNN-Bi-LSTM

## Abstract

The center of pressure (CoP) is a key biomechanical indicator for assessing balance and fall risk; however, force plates, the gold standard for CoP measurement, are costly and impractical for widespread use. Low-cost alternatives such as inertial units or pressure sensors are limited by drift, sparse sensor coverage, and directional performance imbalances, with previous supervised learning approaches reporting ML-AP NRMSE differences of 3.2–4.7% using 1D time-series models on sparse sensor arrays. Therefore, we propose a tactile sensor-based CoP estimation system using deep learning models that can extract 2D spatial features from each pressure distribution image with CNN/ResNet encoders followed by a Bi-LSTM for temporal patterns. Using data from 23 healthy adults performing four balance protocols, we compared ResNet-Bi-LSTM and CNN-Bi-LSTM with baseline CNN-LSTM and Bi-LSTM models used in previous studies. Model performance was validated using leave-one-out cross-validation (LOOCV) and evaluated with RMSE, NRMSE, and R^2^. The ResNet-Bi-LSTM with angular features achieved the best performance, with RMSE values of 18.63 ± 4.57 mm in the mediolateral (ML) direction and 17.65 ± 3.48 mm in the anteroposterior (AP) direction, while reducing the ML/AP NRMSE difference to 1.3% compared to 3.2–4.7% in previous studies. Under dynamic protocols, ResNet-Bi-LSTM maintained the lowest RMSE across models. These findings suggest that tactile sensor-based systems may provide a cost-effective alternative to force plates and hold potential for applications in gait analysis and real-time balance monitoring. Future work will validate clinical applicability in patient populations and explore real-time implementation.

## 1. Introduction

As the aging population rapidly grows worldwide, the fall problem of the elderly is emerging as a serious public health issue. According to the World Health Organization (WHO), approximately 16% of the world’s population is expected to be over the age of 60 by 2030, resulting in a rapid increase in social burdens, including health problems and increased medical expenses caused by falls [1,2,3]. Falls are a major cause of poor quality of life, as well as fractures, long-term disabilities, and mobility restrictions, and the importance of balance assessment to prevent them is gradually increasing [4].

In evaluating balance state, the center of pressure (CoP) is widely used to quantitatively assess the ability to maintain body balance. CoP is calculated based on the magnitude and moment of ground reaction forces and is a key biomechanical indicator for quantitatively assessing postural stability and balance ability. These CoP-based indicators are used to evaluate the relationship between balance ability and fall risk based on the quantitative characteristics of posture sway. They are also used to quantitatively assess the effectiveness of rehabilitation devices [5,6,7,8,9].

The force plate is widely used as a standard device for measuring CoP and can accurately measure ground reaction forces in both vertical and horizontal directions [10]. Force plates are used in research settings due to their high reliability and accuracy. Still, their high cost, installation issues, and mobility constraints limit their use in homes and even clinical environments [11]. As low-cost alternatives to overcome the limitations of force plates, systems using inertial measurement unit (IMU)-based approaches, insole pressure sensor (IPS), and pressure-sensitive mat (PSM) are being actively studied. However, each of the existing systems has its own limitations. The CoP estimation method using IMU has a limitation in that drift errors accumulate over time during the integration of gyroscope signals, resulting in a gradual decrease in tracking accuracy [12,13]. When used to estimate CoP, IPS can produce spurious non-zero readings, for example, under outsole-stiffness effects or during foot-off (swing) phases [14]. PSM can cover large areas and are highly portable, making them practical for clinical and home use [15]. However, PSMs have structural limitations in that they can only measure vertical forces, and commercial PSMs remain limited in accessibility due to their high cost, despite being more affordable than force plates [16].

Accordingly, a supervised learning-based approach has garnered attention in recent studies as a means to estimate CoP with high accuracy, eliminating the need for expensive force plates. It was mainly done based on low-resolution IPS or a small number of FSR sensors [17,18]. A common limitation in these studies is that the CoP trajectory of each foot differs from that of the entire body, as the CoP of each foot is calculated independently. While Choi et al. (2019) proposed a neural network model that utilizes commercial IPS to transform the CoP of both feet into a unified coordinate system, they demonstrated noticeable differences in the CoP estimation performance between mediolateral (ML) and anteroposterior (AP) directions [19]. This directional imbalance has also been observed in the FSR-based study by Duong et al. (2023) [18]. Furthermore, although this study attempted to convert the CoP of each foot into an integrated coordinate system, there was a limitation in that the CoP range in the ML direction was simply assumed to be half the shoe size [18]. Since these supervised learning-based studies have dealt with data in the form of one-dimensional time series with a relatively small number of sensors, relatively simple model architectures such as 1D CNN-LSTM and Bi-LSTM have been applied. To reduce the estimation gap along ML/APs and to more accurately integrate the body CoP into a unified coordinate system, a novel algorithm-based CoP estimation system is needed that can provide rich spatial information.

The tactile sensor is cheaper than commercial PSMs and has the capability to measure high-resolution pressure distribution data [20]. The tactile sensor is composed of a commercial piezoresistive film with electrodes (copper wires) arranged in a grid pattern on both sides, enabling the output of pressure distribution data in a 2D image format. Previous studies that employed PSM to enhance CoP estimation accuracy have mainly focused on modeling or correction-based approaches. For instance, geometric optimization of sensor layouts has been proposed to maximize CoP accuracy in low-resolution designs by selectively increasing sensor density in high-usage areas [15]. Meanwhile, mathematical compensation models for material properties, such as creep and hysteresis effects, have been developed [21]. In contrast, supervised learning approaches that directly optimize CoP estimation from pressure sensor data remain underexplored.

Therefore, in this paper, we propose a tactile sensor-based CoP estimation system using supervised deep learning models. To utilize the spatial patterns of 2D pressure distribution images, we developed AI models (ResNet-Bi-LSTM, CNN-Bi-LSTM) to estimate body CoP by extracting 2D spatial features from each pressure distribution image through CNN/ResNet encoders followed by a Bi-LSTM decoder to capture temporal patterns of these spatial features. Additionally, we incorporated two extra input features. First, we utilized CoP coordinates calculated by the weighted mean approach (WMA) method as a complementary input feature, with the performance of static object CoP estimation demonstrated in previous studies [22]. Second, based on previous studies [23,24] that show spatio-temporal changes in CoP are closely related to lower-limb joint movement, we employed a multimodal approach that additionally uses angle, angular velocity, and angular acceleration information of the lower-limb joints measured by low-cost RGB cameras and analyzed their effects together. To demonstrate that the proposed body CoP estimation models (ResNet-Bi-LSTM, CNN-Bi-LSTM) are most suitable for tactile sensor-based systems, we compared their performance with models used in existing FSR-based CoP estimation studies (CNN-LSTM, Bi-LSTM). 25 healthy adults were recruited for this study. After excluding 2 participants due to force plate measurement errors, data from 23 participants performing four types of balance protocols were used for model development and validation.

Through this approach, we expect that the proposed system could provide a practical CoP monitoring solution that can be efficiently utilized in clinical settings or general households by implementing an accessible balance measurement system that can replace expensive force plates through AI-enhanced low-cost tactile sensors and RGB cameras.

The contributions of this study address three critical limitations identified in existing CoP estimation literature. First, previous supervised learning approaches have utilized sparse sensor configurations with 1D time-series models [17,18,19], which cannot fully exploit the spatial information available in pressure distribution data. We propose a novel framework employing 2D CNN/ResNet encoders combined with LSTM/Bi-LSTM decoders specifically designed for high-resolution (64 × 64) tactile sensors, enabling extraction of rich 2D spatial features from pressure images. Second, prior IPS-based studies have reported significant directional performance imbalances between ML and AP directions, with NRMSE differences ranging from 3.2% to 4.7% [18,19]. We introduce a multimodal learning approach that integrates tactile sensor data with lower-limb kinematic features from RGB-based 3D pose estimation to substantially mitigate this imbalance. Third, unlike previous work focusing on individual foot CoP [17] or making simplified ML range assumptions [18], we present a unified body CoP estimation system. Through systematic evaluation of four architectures across both static and dynamic protocols, we demonstrate the feasibility of tactile sensor-based systems as cost-effective alternatives to force plates, with broader applicability for clinical and home-based balance monitoring.

## 2. Materials and Methods

### 2.1. System Overview and Experimental Environment for CoP Estimation and Evaluation

**Figure 1:**
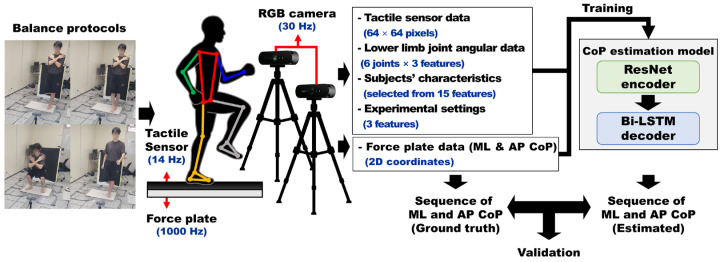
Overview of the CoP estimation and validation pipeline: Pressure image data, lower-limb joint angular data (angle, angular velocity, angular acceleration of hip, knee, and ankle), subjects’ characteristics and experimental settings (protocol ID, physical width and length of tactile sensor) are used as inputs to ResNet-Bi-LSTM model to estimate mediolateral (ML) and anteroposterior (AP) CoP trajectories during balance protocols (one leg stance, tandem stance, squat and walking-in-place). The estimated CoP trajectories are compared against CoP trajectories obtained from the 6-axis force plate.

Our study proposes a data-driven approach for estimating the body CoP trajectories during various balance tasks based on high-resolution tactile sensor data and lower-limb angular data. As shown in Figure 1, subjects performed four types of balance protocols (one leg stance, tandem stance, squat, walking-in-place) while standing on a custom-built tactile sensor placed over two 6-axis force plates.

Synchronized data were collected from three sources: (1) tactile sensor arrays capturing pressure image sequences, (2) a pair of synchronized RGB cameras (Brio 4K, Logitech, Lausanne, Switzerland) used to estimate lower-limb joint angles through MediaPipe Pose [25]-based 3D human pose estimation, and (3) force plates serving as the gold-standard device for acquiring CoP data. The force plate-derived CoP was used as the ground truth label for training and validating the model.

The pressure images acquired from the tactile sensor were used as input features for the CoP estimation neural network models. Additionally, the CoP computed using the WMA from these pressure images was also used as an input feature for model training. Six anthropometric and body composition variables (weight, gender, lean mass, upper limb length, lower-limb length, and waist-hip ratio) were measured and incorporated as subjects’ characteristics. Experimental settings, including protocol ID and the physical dimensions of the tactile sensor (sensor width and height), were also included as additional inputs. From the RGB-based 3D skeleton, lower-limb joint kinematic features—including angles, angular velocities, and angular accelerations of the hip, knee, and ankle—were computed.

All of these input features were used to train neural network models (ResNet-Bi-LSTM, CNN-Bi-LSTM, CNN-LSTM, and Bi-LSTM) to estimate the ML and AP CoP trajectories. The estimated CoP values were quantitatively compared against the reference CoP values obtained from the force plate to evaluate and compare the performance of each model.

### 2.2. System Configuration and Data Acquisition

#### 2.2.1. Tactile Sensor

**Figure 2:**
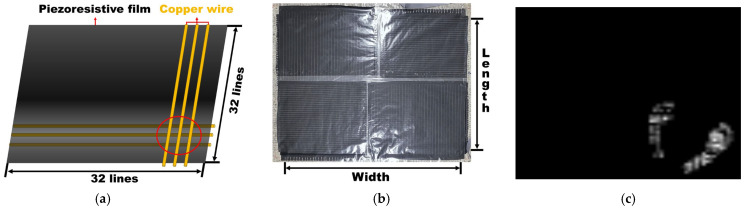
(**a**) The internal orthogonal structure of copper wires on both sides of the piezoresistive film. The red circle highlights the intersection forming an individual sensing point. (**b**) Tactile sensor (80 cm (length) × 60 cm (width)). (**c**) Tactile sensor response during standing.

To estimate the body CoP, pressure image data were collected from tactile sensors by measuring the pressure values at each sensor cell. As shown in Figure 2a, each sensor module consists of a 32 × 32 grid and is capable of measuring pressure up to 14 kPa with the highest sensitivity of 0.3 kPa through resistance changes at the intersections of orthogonally arranged electrodes on both sides of a commercial piezoresistive film [20,26].

This piezoresistive tactile sensor was fabricated in-house using low-cost commercial materials. Specifically, it was constructed using Velostat 1704 piezoresistive film (purchased at $165 for 0.9 m × 45 m) and copper wire (purchased at $4.2 for 0.2 mm × 100 m). Since this is a custom-fabricated sensor utilizing commercial components, the sensor specification (such as size and resolution) can be easily controlled by adjusting the dimensions of the piezoresistive film or spacing of the copper wire placement. The sensing board can be either custom-made or ordered through a PCB manufacturing company using publicly available design files (e.g., https://yyueluo.com/tactile-skin-tool/index.html (accessed on 11 December 2025)). Ordering the board typically incurs a cost of approximately $200, whereas fabricating it in-house can further reduce the overall cost.

Therefore, this system results in an approximate cost of $206 per sensor module. This cost is significantly lower compared to the force plate we used, which was priced at $19,000 per module. Other commercially available force plates of a similar size, such as the KINVENT K-DELTAS and Hawkin Dynamics systems, are also priced in the range of $6000–$7000 plus software fees. Furthermore, in terms of portability and convenience, our sensing system is remarkably lightweight, weighing less than 1 kg (including the thin mat and the board), and is flexible. This contrasts with those commercial force plates, which weigh between 8 kg and 18.5 kg, thus offering considerable advantages for storage and transportation [20].

As illustrated in Figure 2b, four such modules, each measuring 40 cm × 30 cm, were combined to form a 64 × 64 grid, covering an area of approximately 80 cm (length) × 60 cm (width) to capture the pressure distribution of the feet. Figure 2c shows an example of sensor responses when a person stands on the tactile sensor. The high-resolution tactile sensor inherently captures individual variations in foot contact area and pressure distribution patterns, reflecting differences in foot size and morphology across participants.

The raw tactile sensor data were collected by integrating four individual tactile sensor modules into a unified 64 × 64 sensor array. As each module may exhibit different baseline values, a calibration process was performed to correct these initial discrepancies. Although no significant change was observed during the current experiments, the baseline value of the piezoresistive tactile sensors may change over time [27]. To prevent this, we collected 20 frames of sensor data with nothing placed on the sensor before every experiment. The measured value of each cell was then averaged to define a 64 × 64 baseline value. In the real-time main measurements, the measured data was corrected by subtracting this baseline value to obtain the true sensor response. Additionally, values below a certain threshold were set to zero, which ensured that only data corresponding to substantial pressure was collected. To further ensure data consistency, especially against potential decreases in sensor sensitivity (i.e., a rise in the baseline value), the entire collected dataset was scaled to a [0,1] range using Min-Max normalization to ensure the sensor can capture the same data even if the sensor sensitivity has been slightly changed. Then we convert this sensor data into 8-bit grayscale images by multiplying by 255 [22]. To eliminate residual artifacts such as salt-and-pepper noise, a previously validated denoising algorithm was applied as in prior work [22].

In this study, although the WMA was not employed as the primary method for estimating the CoP, CoP values derived from tactile sensor data using WMA were used as auxiliary input features to enhance the models’ learning capacity. These CoP values were computed by applying a weighted average formula to the pressure values and their corresponding spatial coordinates ($x_{i}$, $y_{i}$) across the 64 × 64 tactile sensor array, as shown in Equation (1):

(1) $${CoP}_{ML}=\frac{\sum_{i=1}^{64}x_{i}w_{i}}{\sum_{i=1}^{64}w_{i}}\times \frac{L}{64}{,\;CoP}_{AP}=\frac{\sum_{i=1}^{64}y_{i}w_{i}}{\sum_{i=1}^{64}w_{i}}\times \frac{W}{64}$$

where $w_{i}$ denotes the pressure value at position ($x_{i}$, $y_{i}$), L and W represent the physical length and width of the tactile sensor, respectively.

#### 2.2.2. 3D Pose-Based Joint Angular Feature Extraction

Two RGB cameras were positioned in a stereo configuration at approximately 2 m from participants to capture full-body movements for 3D human pose estimation. Frame acquisition was synchronized through multi-threaded implementation, where both camera streams were initialized concurrently and read frame-by-frame in lockstep, ensuring corresponding frames were captured at effectively identical timestamps.

Stereo camera calibration was performed using a checkerboard pattern with MATLAB R2022b Stereo Camera Calibrator to obtain intrinsic and extrinsic parameters, from which projection matrices were computed for Direct Linear Transformation (DLT). MediaPipe Pose was applied to extract 2D skeletal keypoints from the captured images [25].

Although MediaPipe Pose provides 3D pose estimations from a single RGB camera, its accuracy along the *z*-axis (depth) is limited, and the reliability of keypoint detection decreases significantly in the presence of occlusion. To address these limitations, we extracted 2D keypoints independently from both calibrated camera views and performed DLT-based triangulation on corresponding keypoints to reconstruct accurate 3D human poses [28,29]. The implementation followed established methods (https://github.com/TemugeB/bodypose3d (accessed on 11 December 2025)). For each landmark, if valid 2D coordinates were detected in both views, the DLT algorithm triangulated the 3D position. Calibration quality and reconstruction accuracy were maintained by fixing camera positions throughout all data collection sessions and monitoring pose tracking quality through visualization during data preprocessing.

Subsequently, angles for the lower-limb joints (hip, knee, and ankle) on both legs were calculated using the vector dot product method. Each joint angle was defined as the angle between two vectors formed by adjacent keypoints. Joint angular velocities and accelerations were obtained by computing the first and second temporal derivatives of the joint angles, respectively.

#### 2.2.3. Synchronized Data Collection and Post-Processing

Two Bertec FP4060-08-TM force plates (Bertec Corporation, Columbus, OH, USA) were used to measure ground reaction forces and moments (F_X_, F_Y_, F_Z_, M_X_, M_Y_, M_Z_) at a sampling rate of 1000 Hz. The CoP values were directly computed using Bertec Device Controller software (v1.1.0.1302) and served as the ground truth for model training and validation. To ensure precise temporal alignment across sensor modalities, all collected sensor data were synchronized through post-processing using downsampling and interpolation techniques. Ground truth CoP data (1000 Hz) from Bertec Device Controller software and RGB image data (30 Hz) were downsampled to 14 Hz, while tactile sensor data (14 Hz) were resampled through linear interpolation to ensure temporal consistency, creating a unified multimodal dataset at a 14 Hz sampling rate. Data collection across all sensors was synchronized using a manual trigger (keyboard input) to ensure simultaneous initiation and termination.

#### 2.2.4. Data Synchronization & Sampling

A unified sampling rate of 14 Hz was selected because it matches the native acquisition speed of the tactile sensor, ensuring that all modalities can be aligned without exceeding its hardware limitations. Since manual triggers are stored using each device’s frame-level timestamp—meaning the event is registered at the timestamp of the nearest acquired frame rather than at the exact press time—the expected synchronization uncertainty is bounded by half of their sampling intervals (71 ms for the tactile sensor and 33 ms for the RGB camera), resulting in an estimated mismatch of approximately 52 ms.

### 2.3. Experimental Protocol

#### 2.3.1. Participants

25 healthy adults were initially recruited for this study. 2 participants were excluded because the commercial force plate experienced data logging errors during measurement. Consequently, data from 23 participants (15 males and 8 females; age: 22.7 ± 1.6 years, height: 171.6 ± 7.8 cm, weight: 73.4 ± 13.5 kg) were used to construct the training and validation dataset for the deep learning models. The study was approved by the Institutional Review Board of Gyeongsang National University (GNU IRB approval number: GIRB-B25-NY-0016) and was conducted in accordance with the Declaration of Helsinki. All subjects reported no history of neurological, musculoskeletal, or vestibular disorders and provided written informed consent before participation.

#### 2.3.2. Protocol

**Figure 3:**
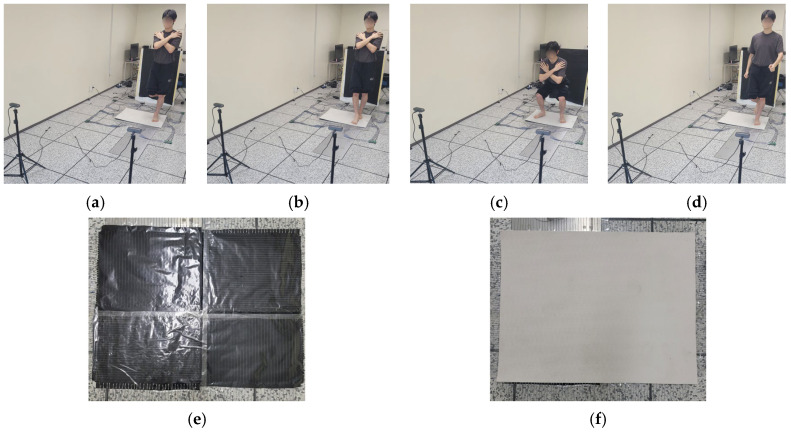
The balance tasks and tactile sensor surface with/without the soft silicone mat. (**a**) one leg stance, (**b**) tandem stance, (**c**) squat, (**d**) walking-in-place, (**e**) tactile sensor surface without the soft silicone mat, (**f**) tactile sensor surface with the soft silicone mat.

As shown in Figure 3, the subjects were asked to perform four commonly used tasks as part of the balance assessment protocol: one-leg stance, tandem stance, squat, and walking-in-place. For static balance evaluation, the one-leg stance and tandem stance tasks were selected, while the squat and walking-in-place tasks represented dynamic conditions. In the one-leg stance, subjects stood on their dominant foot, maintaining balance with the non-dominant foot lifted. In the tandem stance, the dominant foot was placed in front of the non-dominant foot, such that the toe of the rear foot touched the heel of the forward foot. The rationale for selecting these specific balance protocols is discussed in Section 5.

During the squat task, subjects performed controlled squatting motions at a consistent rate of one repetition every 3 s. In the walking-in-place task, subjects were instructed to walk on the spot at a self-selected, consistent pace. All balance tasks were performed barefoot. Except for the walking-in-place task, subjects were instructed to keep their arms crossed over their chests during all other tasks. Foot dominance was determined based on the subjects’ preferred kicking foot [30].

To ensure data reliability, each of the four tasks was performed twice, with a 30-s rest between repetitions of the same task and a 60-s rest between different tasks, resulting in a total of 8 trials per subject. Each trial lasted 90 s, with data from the middle 80 s used for dataset construction. As shown in Figure 3, all tasks were conducted on a 10 mm thick silicone form mat placed over the tactile sensor surface. The use of this yoga-mat–like soft silicone mat maintained the sensor’s pressure-measurement performance while simultaneously providing comfort to the user [20]. This approach follows a previous tactile sensor–based study that successfully implemented the same mat thickness without compromising detection stability [20].

### 2.4. Data Preprocessing and Feature Engineering

#### 2.4.1. Data Preprocessing

The preprocessing pipeline was initiated based on manually recorded spacebar signals, marking the start and end points of the experiment. Subsequently, both RGB image sequences and tactile sensor data were segmented into valid intervals corresponding to the active recording phases.

From the RGB images, 3D human pose data were extracted using MediaPipe Pose, which estimates 33 anatomical keypoints per frame. To remove high-frequency noise, the extracted 3D keypoint trajectories were filtered using a zero-phase fourth-order Butterworth low-pass filter with a cutoff frequency of 2 Hz [28]. The tactile sensor data consisted of consecutive pressure images with a spatial resolution of 64 × 64. Linear interpolation was applied when frame drops occurred to maintain temporal continuity and ensure consistency in the sequential dataset [31]. The ground truth CoP from the force plate signals was filtered with a zero-phase fourth-order Butterworth low-pass filter with a cutoff frequency of 10 Hz [32].

Power spectral density (PSD) analysis of the original 1000 Hz force plate signals confirmed that more than 99.7% of the total spectral energy remained below 7 Hz after applying a 10 Hz low-pass Butterworth filter, indicating that aliasing is negligible when resampling to 14 Hz. Accordingly, all datasets—including tactile sensor data, WMA CoP, ground truth CoP, and 3D keypoints—were resampled to 14 Hz using a common temporal reference, where the timestamps of the downsampled force plate signals served as the alignment baseline for all modalities.

Tactile sensor data and 3D keypoints were then resampled via interpolation to align precisely with these timestamps. The WMA CoP was calculated for each tactile sensor frame using Equation (1). Subsequently, the temporally synchronized 3D keypoints served as the basis for extracting joint-specific kinematic features—namely, joint angles, angular velocities, and angular accelerations—at the hip, knee, and ankle joints.

#### 2.4.2. Input Feature Construction

For each trial, the following were consolidated into a single HDF5 file:

- Tactile pressure images (64 × 64)
- Tactile WMA CoP data
- Ground truth CoP data
- Lower-limb angular features (angles, angular velocities, and angular accelerations for the hip, knee, and ankle joints)
- Subjects’ characteristics
- Experimental settings

Subjects’ characteristics included personal information (PI), bioelectrical impedance analysis (BIA), and manual anthropometry (MA). PI consisted of gender and age recorded during participant enrollment. BIA values were measured using an InBody device (H30N, InBody, Seoul, Republic of Korea), while MA and experimental settings were obtained through manual measurements. All collected input variables are summarized in Table 1.

## 3. CoP Estimation Algorithm

In this study, four deep learning models were employed for CoP estimation, including ResNet-Bi-LSTM, CNN-Bi-LSTM, CNN-LSTM, and Bi-LSTM. The CoP estimation process for each model was as follows. Each model took as input sequences of 64 × 64 pressure images. For each frame, the model predicted a two-dimensional CoP coordinate (CoP_ML_, CoP_AP_). To enhance the predictive performance, additional features were concatenated to the pressure image features within the model, including the tactile WMA CoP calculated from the pressure images using Equation (1), lower-limb angular features, subjects’ characteristics, and experimental settings.

The ground truth for CoP at each frame was derived from force plate measurements, ensuring accurate correspondence between model predictions and reference values. Model training aimed to minimize the mean squared error (MSE) between predicted CoP and ground truth CoP, with the *x*-axis representing the ML direction and the *y*-axis representing the AP direction.

**Figure 4:**
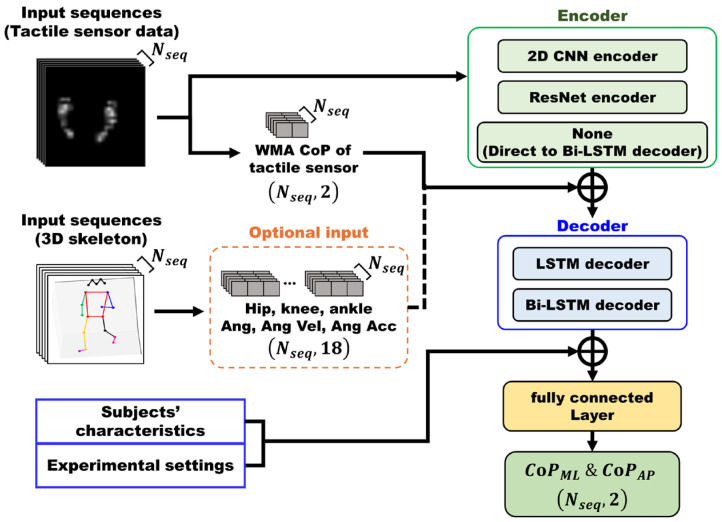
Overview of CoP estimation model architectures.

**Figure A1:**
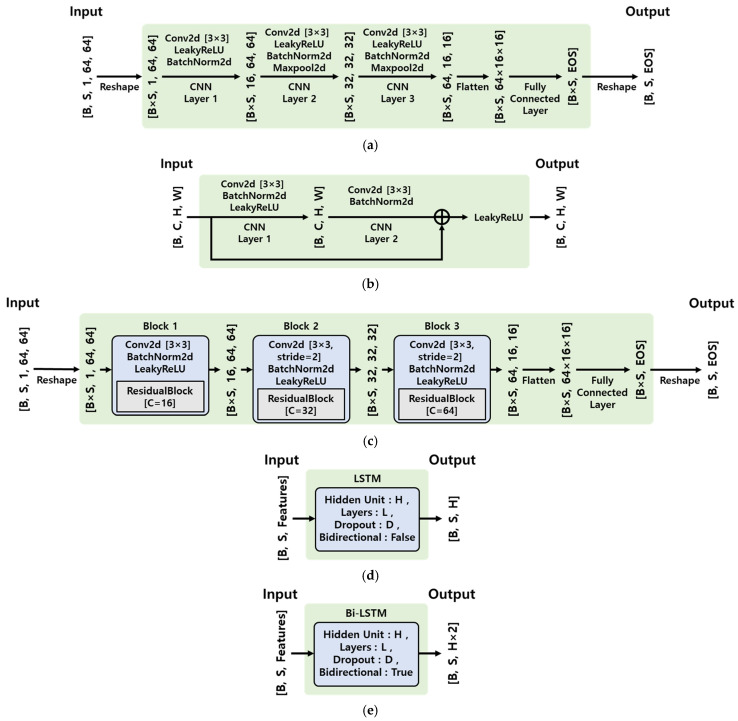
Detailed architectures of deep learning model components. (**a**) CNN Encoder, (**b**) Residual Block, (**c**) ResNet Encoder, (**d**) LSTM Decoder, (**e**) Bi-LSTM Decoder. All convolutions use 3 × 3 kernels with padding = 1 unless stride is specified. B: Batch Size; S: Sequence Length; EOS: Encoder Output Size; H: Image Height; W: Image Width; C: Channels.

Additionally, to investigate the impact of lower-limb angular features on CoP estimation performance, model training and validation were conducted separately for two cases: one with angular data and one without. Figure 4 illustrates the overall architecture framework, showing how the four models are constructed through different encoder–decoder combinations. Detailed implementation specifications for each architecture are presented in Figure A1.

### 3.1. Encoder Architecture

To effectively extract spatial features from tactile pressure sequences, three distinct encoder configurations were implemented and evaluated. Previous studies have demonstrated that CNN-Bi-LSTM architectures show superior performance in applications involving sequential visual data, including breast cancer diagnosis, gold price forecasting, and livestock behavior classifications [33,34,35], which motivated our encoder design choices.

The 2D CNN encoder processes 64 × 64 tactile pressure images through multiple convolutional layers, leveraging 2D convolutional operations to capture local patterns and spatial relationships within the pressure distribution. This architecture enables the model to extract essential spatial features required for precise CoP estimation.

To enhance spatial feature extraction capability, the ResNet encoder incorporates residual connections that enable practical training of deeper networks while mitigating the vanishing gradient problem [36,37]. Unlike the standard CNN encoder, which stacks convolutional layers sequentially, this ResNet encoder facilitates learning of more expressive spatial features. To preserve spatial information, all pooling layers were replaced with strided convolutional layers, enabling learnable downsampling while maintaining detailed contextual information for CoP prediction.

In contrast, the no encoder configuration directly flattens the 64 × 64 tactile pressure images and feeds them into the Bi-LSTM decoder without any convolutional feature extraction. This approach served as a baseline for evaluating the contribution of spatial feature extraction, allowing direct comparison of the decoder’s performance with and without encoded spatial representations.

### 3.2. Decoder Architecture

After the encoder stage, two decoder configurations were implemented to model temporal dependencies in the tactile pressure sequences. The decoder selection was motivated by a previous CoP estimation study that demonstrated the effectiveness of the CNN-LSTM architecture [17].

The LSTM decoder processed the encoder output through unidirectional recurrent layers to capture temporal dependencies within the sequence. This configuration enabled effective modeling of the sequential tactile pressure patterns for CoP prediction. In contrast, the Bi-LSTM decoder employed bidirectional processing, capturing information from both past and future contexts simultaneously [18,38]. Unlike conventional LSTM, the Bi-LSTM processes sequences in both forward and backward directions, enabling more comprehensive modeling of temporal dependencies.

### 3.3. Feature Integration and CoP Estimation

Multiple types of additional information were integrated at different stages to enhance CoP estimation accuracy. Following the encoder stage, two categories of additional features were concatenated with the spatial features extracted from the encoder before being processed by the decoder. The first category consisted of WMA CoP values calculated from the pressure images of the tactile sensor using Equation (1). These features provided complementary spatial information that supplemented the encoder’s output. The second category consisted of lower-limb joint angular kinematic data, which were selectively incorporated as input features for ablation studies to evaluate the contribution of kinematic information to CoP estimation performance.

The augmented features from the encoder output, along with these additional inputs, were then processed by the decoder to learn temporal patterns in the sequence. Following the decoder stage, subjects’ characteristics and experimental settings were concatenated with the decoder output to provide additional information for accurate CoP estimation. The final concatenated feature vector was processed through fully connected layers to produce CoP trajectories in both ML and AP directions as the output of the overall architecture.

### 3.4. Hyperparameter Optimization

Hyperparameter tuning is a critical step in deep learning, as it directly influences the generalization capability of the model across diverse input data. However, the tuning process is computationally expensive and time-consuming, especially as the number and range of hyperparameters increase. To manage this complexity, we constrained the search space by limiting both the number of hyperparameters and their value ranges. We applied grid search with identical parameter ranges across all models to ensure objective performance comparisons, allowing us to identify the most effective configuration within practical training time [39,40,41]. Additionally, a StepLR learning rate scheduler was employed during training to enhance convergence stability. Hyperparameters for each model were optimized within the value ranges presented in Table A1.

### 3.5. Model Evaluation

The models were evaluated using the LOOCV method, which ensures that each subject serves once as the validation dataset while the remaining subjects form the training set. This approach minimizes bias and enables a robust assessment of generalization performance [18,42]. RMSE, NRMSE, and R^2^ were employed as the evaluation metrics to quantitatively assess the prediction accuracy of each model, as described in Equations (2)–(4):

(2) $$RMSE=\sqrt{\frac{\sum_{i=1}^{n}{\left(y_{i}-{\hat{y}}_{i}\right)}^{2}}{n}}$$

(3) $$NRMSE=\frac{1}{y_{max}-y_{min}}\sqrt{\frac{\sum_{i=1}^{n}{\left(y_{i}-{\hat{y}}_{i}\right)}^{2}}{n}}\times 100$$

(4) $$R^{2}=1-\frac{\sum_{i=1}^{n}{\left(y_{i}-{\hat{y}}_{i}\right)}^{2}}{\sum_{i=1}^{n}{\left(y_{i}-\bar{y}\right)}^{2}}$$

where $y_{i}$ represents the ground truth CoP value, ${\hat{y}}_{i}$ represents the estimated CoP value, n represents the total number of estimations across all validation data, and $y_{max}$ and $y_{min}$ represent the maximum and minimum values of the ground truth CoP, respectively.

### 3.6. Statistical Analysis

To evaluate the effects of model architecture and balance condition on CoP estimation performance, a two-way repeated-measures ANOVA (RMANOVA) was conducted for each direction (ML, AP). The two within-subject factors were Model (Bi-LSTM, CNN-LSTM, CNN-Bi-LSTM, ResNet-Bi-LSTM) and Input Condition.

Input Condition consisted of two settings:

- with angular data (WA), where lower-limb angular features were included
- only tactile data (OT), where the model used tactile sensor input alone

The dependent variable was the RMSE for each direction (ML, AP) between the estimated CoP and the ground truth CoP. Statistical analyses were performed using Python 3.10 with Pingouin (v 0.5.3) for RMANOVA.

## 4. Results

**Figure 5:**
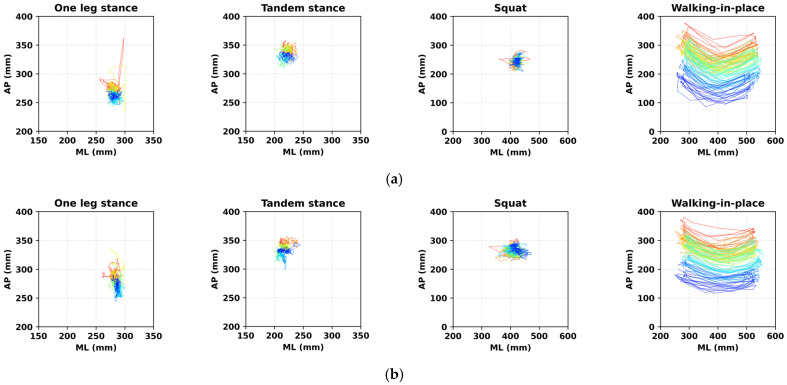
Stabilograms of a representative subject recorded under different balance protocols. The red-to-blue color transition represents the trial time from start to end. (**a**) Ground truth stabilograms from force plates. (**b**) Estimated stabilograms from the ResNet-Bi-LSTM model in the WA condition.

**Figure 6:**
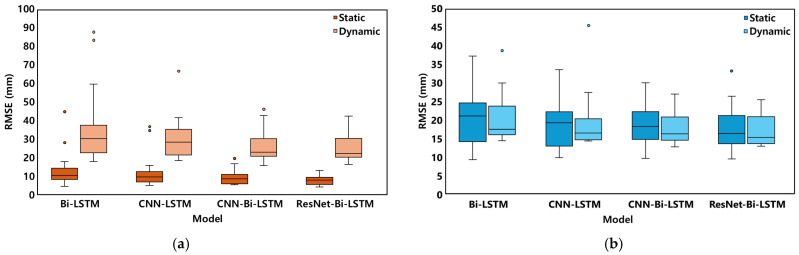
Boxplot of RMSE in ML/AP directions for each AI model under overall balance protocols (**a**) RMSE(ML), (**b**) RMSE(AP).

**Figure 7:**
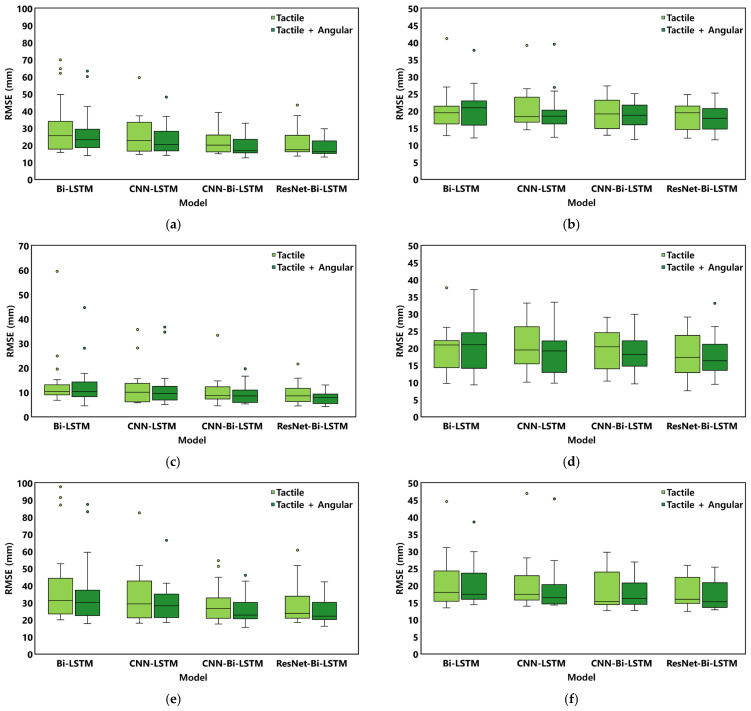
Boxplot of experimental results comparing tactile + angular input and tactile-only input (**a**) RMSE(ML) under overall protocols, (**b**) RMSE(AP) under overall protocols, (**c**) RMSE(ML) under static protocols, (**d**) RMSE(AP) under static protocols, (**e**) RMSE(ML) under dynamic protocols, (**f**) RMSE(AP) under dynamic protocols.

**Figure 8:**
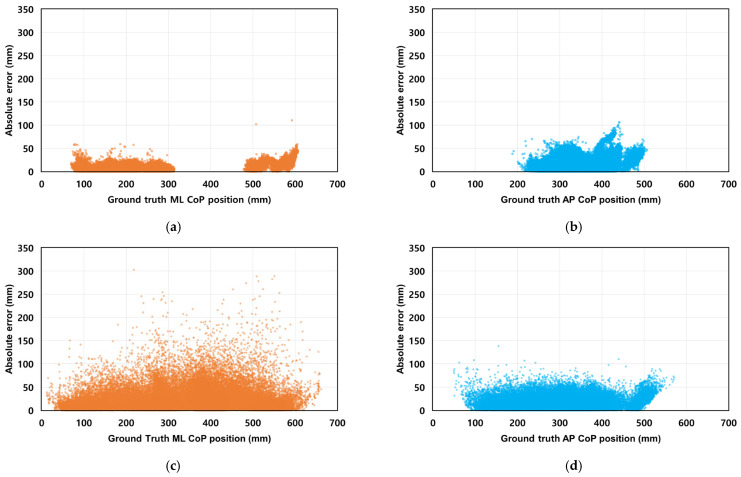
Scatterplot of absolute error distribution by ground truth CoP position for the ResNet-Bi-LSTM model under different protocols (**a**) Absolute error (ML) under static protocols, (**b**) Absolute error (AP) under static protocols, (**c**) Absolute error (ML) under dynamic protocols, (**d**) Absolute error (AP) under dynamic protocols.

Stabilograms for a representative subject under each balance protocol, including both ground truth and estimated trajectories, are shown in Figure 5. The model performance was evaluated for the overall, static, and dynamic protocols, and the results are summarized in Table 2 and Table 3, and Figure 6, Figure 7 and Figure 8. In addition, the statistical comparisons of the model architectures and input conditions are reported in Table 4. The final optimal combination of hyperparameters used in each model training is presented in Table A2.

The CoP estimation performance according to the input data composition is presented in Table 2 and Figure 7. Across all model architectures, including lower-limb angular data improved estimation accuracy. When angular data was added (WA condition), the overall RMSE decreased and R^2^ increased compared to using tactile sensor data alone (OT condition) (Table 2). Figure 7 visually illustrates this trend through boxplots for each protocol, demonstrating that the median RMSE for the WA condition is consistently lower than that for the OT condition under all protocol conditions (Figure 7c–f).

The comparison results of model performance according to protocol characteristics are presented in Table 3 and Figure 6. Dynamic balance tasks proved more challenging than static tasks across all models. Under the WA condition, dynamic protocols consistently yielded higher RMSE values than static protocols. ML-direction errors tended to exceed AP-direction errors across most conditions. Among all tested architectures, the ResNet-Bi-LSTM model demonstrated the smallest RMSE increase when transitioning from static to dynamic protocols.

As shown in Table 3, the two-way RMANOVA revealed a significant main effect of Model for both the ML (*p* < 0.01) and AP (*p* < 0.05) directions, indicating that CoP estimation accuracy differed across the four model architectures. A significant main effect of Input Condition was observed in the ML direction (*p* < 0.001), demonstrating that including lower-limb angular data (WA) consistently reduced RMSE compared with using tactile data alone (OT). In contrast, this effect did not reach significance for the AP direction (*p* > 0.05), suggesting that angular information did not significantly improve AP estimation. No significant Model × Input Condition interaction was detected in either direction (ML and AP: *p* > 0.05), indicating that the performance gain from angular data was consistent across all model architectures and did not depend on the specific model type.

The distribution of absolute errors relative to ground truth CoP positions of the ResNet-Bi-LSTM model with the lowest RMSE among the four CoP estimation models is presented in Figure 8. In the static protocols (Figure 8a,b), most errors fell within 50 mm in the ML direction and within 100 mm in the AP direction. In the dynamic protocols (Figure 8c,d), the CoP movement range was larger, and errors were generally greater compared to static protocols.

**Table 2:** Comparison of deep learning models’ performance across all protocol data.

	ResNet-Bi-LSTM		CNN-Bi-LSTM		CNN-LSTM		Bi-LSTM	
Input Data	WA ^1^	OT ^2^	WA ^1^	OT ^2^	WA ^1^	OT ^2^	WA ^1^	OT ^2^
ML RMSE (mm) (SD)	**18.63** (4.57)	21.84 (7.91)	19.89 (5.51)	22.11 (7.11)	22.95 (8.34)	25.51 (10.58)	27.23 (12.78)	30.23 (15.77)
AP RMSE (mm) (SD)	**17.65** (3.48)	18.26 (3.71)	18.50 (3.50)	19.33 (4.46)	19.42 (5.61)	20.37 (5.32)	20.47 (5.50)	20.16 (5.76)
ML NRMSE (%) (SD)	**4.23** (1.05)	4.90 (1.51)	4.48 (1.04)	4.98 (1.40)	5.17 (1.72)	5.73 (2.14)	6.27 (3.47)	6.88 (3.85)
AP NRMSE (%) (SD)	**5.55** (1.70)	5.72 (1.58)	5.73 (1.19)	5.96 (1.35)	5.96 (1.38)	6.32 (1.60)	6.32 (1.61)	6.23 (1.64)
ML R^2^ (SD)	**0.97** (0.02)	0.96 (0.03)	0.97 (0.02)	0.96 (0.03)	0.96 (0.03)	0.94 (0.05)	0.91 (0.15)	0.90 (0.16)
AP R^2^ (SD)	**0.87** (0.09)	0.86 (0.10)	0.86 (0.08)	0.85 (0.10)	0.85 (0.09)	0.83 (0.11)	0.84 (0.09)	0.84 (0.10)

^1^ With lower-limb angular feature data, ^2^ only tactile sensor data.

**Table 3:** Comparison of deep learning models’ performance across static and dynamic protocol data with lower-limb angular feature data.

	ResNet-Bi-LSTM		CNN-Bi-LSTM		CNN-LSTM		Bi-LSTM	
Protocol	Static	Dynamic	Static	Dynamic	Static	Dynamic	Static	Dynamic
ML RMSE (mm) (SD)	**8.09** (3.24)	**24.88** (6.36)	8.95 (3.70)	26.32 (7.91)	11.54 (7.98)	29.55 (10.71)	12.97 (8.23)	35.21 (18.00)
AP RMSE (mm) (SD)	**17.47** (5.71)	**17.13** (3.91)	18.90 (5.09)	17.55 (3.93)	19.24 (6.63)	18.97 (6.70)	20.04 (6.45)	20.54 (5.78)
ML NRMSE (%) (SD)	**6.36** (2.97)	**6.51** (1.65)	7.15 (3.94)	6.83 (1.86)	8.33 (4.25)	7.76 (3.14)	10.33 (6.26)	9.29 (5.47)
AP NRMSE (%) (SD)	**12.66** (6.56)	**5.53** (1.15)	13.83 (7.03)	5.65 (1.07)	13.54 (6.56)	6.05 (1.56)	14.01 (5.73)	6.59 (1.49)
ML R^2^ (SD)	**0.89** (0.18)	**0.92** (0.05)	0.83 (0.28)	0.91 (0.05)	0.81 (0.27)	0.88 (0.12)	0.62 (0.73)	0.79 (0.36)
AP R^2^ (SD)	**0.42** (0.89)	**0.89** (0.08)	0.26 (1.22)	0.89 (0.07)	0.23 (1.44)	0.87 (0.10)	0.36 (0.94)	0.85 (0.10)

**Table 4:** *p*-Values of the two-way RMANOVA—model(M), input condition(IC).

	M ^1^	IC ^2^	M ^1^ × IC ^2^
ML RMSE	0.002	<0.001	0.722
AP RMSE	0.022	0.102	0.132

^1^ Model (Bi-LSTM vs. CNN-LSTM vs. CNN-Bi-LSTM vs. ResNet-Bi-LSTM), ^2^ Input Condition (WA vs. OT).

## 5. Discussion

In this study, we aimed to develop a cost-effective alternative to force plates for body CoP estimation by integrating high-resolution tactile sensor data with deep learning models. We systematically compared four deep learning architectures (ResNet-Bi-LSTM, CNN-Bi-LSTM, CNN-LSTM, and Bi-LSTM) to evaluate their effectiveness in extracting spatial features from 2D pressure distribution images and modeling temporal patterns for accurate CoP prediction across various balance protocols.

The selection of balance protocols was based on established clinical assessment standards that represent diverse postural control challenges. Tandem stance and one-leg stance protocols were chosen as they constitute fundamental static balance assessments widely used in clinical practice [6,43,44]. Walking-in-place and squat protocols were selected to evaluate dynamic balance capabilities, as these represent standardized dynamic movement assessments in clinical balance evaluation [45,46]. These protocols enabled a comprehensive assessment of model performance across balance-related movements that are commonly encountered in clinical and daily living contexts.

A key finding of this study was the reduction in the directional performance imbalance in CoP estimation to 1.3%, compared to the 3.2–4.7% differences reported in previous studies. This achievement was made possible by incorporating lower-limb angular data (the WA condition), which improved ML direction performance by approximately 10–15% across all models, with ResNet-Bi-LSTM showing the largest improvement of 14.7%. In the AP direction, CNN-based models demonstrated improvements of 3.3–4.7%, while ResNet-Bi-LSTM showed a modest 3.3% improvement but still presented the lowest RMSE (Table 2, Figure 7). This multimodal approach effectively addressed the ML/AP directional performance imbalance reported in previous studies. Previous IPS-based studies reported directional performance imbalances, with NRMSE differences between ML and AP directions of approximately 4.7% (Choi et al., 2019) [19] and 3.2% (Duong et al., 2023) [18]. Such an imbalance can compromise the reliable evaluation of multidirectional postural control, making it essential to achieve balanced accuracy across both directions. Therefore, in this study, the ML RMSE—AP RMSE difference was calculated to quantitatively assess whether the proposed ResNet-Bi-LSTM effectively reduced this imbalance under identical sensor conditions. As shown in Table 2, the WA condition of the ResNet-Bi-LSTM achieved an ML/AP RMSE difference of only 0.98 mm. In contrast, the baseline Bi-LSTM and CNN-LSTM under OT conditions exhibited much larger gaps of 10.07 mm and 5.14 mm, respectively. These findings confirm that the proposed ResNet-Bi-LSTM substantially reduced directional performance imbalances observed in baseline models. This improvement was further corroborated by NRMSE analysis, which showed that the ResNet-Bi-LSTM with WA conditions reduced the ML/AP difference to 1.3%, noticeably lower than the differences reported in previous studies.

This improved directional balance was achieved while maintaining superior overall performance. The results demonstrate that models incorporating 2D spatial feature extraction (CNN-LSTM, CNN-Bi-LSTM, ResNet-Bi-LSTM) consistently outperformed the baseline Bi-LSTM model that relied solely on flattened pressure data (Figure 6). Quantitatively, as shown in Table 2, the ResNet-Bi-LSTM model with WA conditions achieved the lowest RMSE across all protocols, recording 18.63 ± 4.57 mm for the ML direction and 17.65 ± 3.48 mm for the AP direction. This demonstrates that tactile sensors provide richer spatial information compared to the limited sensor arrays used in previous FSR-based studies [17,18], and that 2D convolutional operations represent a more appropriate approach for tactile sensor-based systems than traditional 1D time-series methods.

Protocol-dependent performance variations revealed different patterns across ML and AP directions. In the ML direction, static protocols consistently achieved lower RMSE values than dynamic protocols across all models. For instance, the ResNet-Bi-LSTM model recorded 8.09 ± 3.24 mm (static) versus 24.88 ± 6.36 mm (dynamic). Conversely, AP direction performance remained relatively consistent across protocols, with ResNet-Bi-LSTM showing 17.47 ± 5.71 mm (static) and 17.13 ± 3.91 mm (dynamic). Despite similar RMSE values in the AP direction, NRMSE values differed substantially between protocols, with static protocols showing 12.66 ± 6.56% and dynamic protocols showing 5.53 ± 1.15%. This discrepancy stems from the significantly smaller CoP displacement range in static protocols (318.95 mm) compared to dynamic protocols (521.65 mm), as illustrated in Figure 8. When normalized against these different ranges, similar absolute errors yield higher relative error percentages in static conditions. This phenomenon aligns with findings from Duong et al. (2023) [18], where ML direction showed lower RMSE but higher NRMSE compared to AP direction.

Under dynamic conditions, the ResNet-Bi-LSTM model maintained relatively stable performance compared to the other models. Specifically, in the ML direction, ResNet-Bi-LSTM achieved the lowest RMSE of 24.88 mm, while other models showed higher errors ranging from 26.32 to 35.21 mm. In the AP direction, ResNet-Bi-LSTM also recorded the smallest error of 17.13 mm compared to other models (17.55–20.54 mm). While achieving superior overall performance, the analysis revealed specific patterns under dynamic conditions. In the ML direction, dynamic protocols consistently showed larger RMSE values compared to static protocols across all models (Table 3, Figure 8). These high-error periods likely correspond to critical movement phases involving rapid CoP transitions, such as lateral weight shifts when walking in place. This pattern aligns with previous studies reporting increased RMSE during dynamic CoP estimation tasks [16,47,48,49], confirming that rapid postural changes present consistent challenges across different CoP measurement approaches.

To situate these findings within the broader context of CoP estimation research, Table 5 summarizes representative state-of-the-art approaches, highlighting their sensing modalities, model architectures, and reported ML/AP estimation performance.

This study demonstrates the feasibility of replacing expensive force plates with tactile sensor-based systems for CoP monitoring in both clinical and home environments. The ResNet-Bi-LSTM architecture proved most effective for maintaining stable performance across overall conditions. These results demonstrate the feasibility of tactile sensor-based CoP estimation, which could serve as a foundation for future development of real-time balance monitoring systems with appropriate optimization and validation.

While the proposed system requires RGB cameras, subject characteristics, and experimental settings in addition to the tactile sensor, these requirements are manageable in practice. RGB cameras can be fixed after initial calibration, experimental settings are one-time system parameters configured during setup, and user anthropometric data can be managed through profiles. Considering the lower cost and portability compared to commercial force plates, the initial setup complexity is offset by long-term benefits in accessibility and cost-effectiveness.

However, several limitations remain. First, the proposed approach was evaluated using offline data processing, which may limit its applicability in real-time applications. To address this limitation, we plan to explore the use of JAX, a high-performance computing framework optimized for matrix operations [51]. JAX has been reported to achieve up to 153-fold speedups in computational tasks [51], and given that our deep learning models rely heavily on matrix operations, we anticipate that JAX could significantly reduce processing time and enable real-time implementation in future studies. Regarding sensor drift robustness, similar tactile sensors have demonstrated stability across multiple robustness tests [52,53], and no sensor drift issues were reported even when 20 users performed vigorous exercises on a comparable sensing mat [20]. Therefore, we do not anticipate major concerns regarding short-term robustness. However, long-term stability under sustained loading conditions, such as continuous body weight application, has not been thoroughly investigated and warrants further study. Second, since this study used only data from healthy adults, the generalizability to clinically important populations, such as patients with impaired balance, has not been directly confirmed. Therefore, future research will focus on validation using patient data from clinical groups, as well as real-time system implementation. Second, since this study used only data on healthy adults, the possibility of generalization to clinically important groups such as patients with impaired balance was not directly confirmed. Therefore, for future studies, we will focus on further validation using patient data in clinical groups and real-time implementation.

## 6. Conclusions

In this study, we proposed a tactile sensor-based deep learning framework for estimating the body CoP as a cost-effective alternative to force plates. By integrating high-resolution 2D pressure distribution data with lower-limb kinematic information, the proposed ResNet-Bi-LSTM model demonstrated superior performance compared to conventional CNN-LSTM and Bi-LSTM models in tactile sensor-based CoP estimation, achieving RMSE values of 18.63 ± 4.57 mm (ML) and 17.65 ± 3.48 mm (AP). Notably, while baseline models such as Bi-LSTM and CNN-LSTM exhibited ML/AP directional performance imbalances (10.07 mm and 5.14 mm, respectively), the ResNet-Bi-LSTM reduced this difference to only 0.98 mm, with an NRMSE difference of 1.3%—a clear improvement over values reported in previous studies.

The ResNet-Bi-LSTM further maintained stable performance under dynamic conditions, achieving the lowest errors among all tested architectures (24.88 mm in the ML direction and 17.13 mm in the AP direction). Moreover, tactile sensors can be flexibly configured in terms of their resolution and form, allowing them to be freely changed to the target platforms and application environments [54]. These results demonstrate that tactile sensor-based systems may serve as a cost-effective alternative to force plates and hold potential for applications in gait analysis and real-time balance assessment.

Future research will focus on validating the clinical applicability of this approach using patient data that exhibits balance impairments, as well as on developing real-time implementations for practical use in rehabilitation and healthcare settings.

## Figures and Tables

**Table 1:** All input variables for deep learning.

Subjects’ Characteristics			Tactile Sensor	RGB Camera-Based 3D Pose	Experimental Settings ^7^
PI ^1^	BIA ^2^	MA ^3^			
Gender	Weight	Height	Tactile data	Hip joint angular data (A ^4^/AV ^5^/AA ^6^)	Protocol ID
Age	Skeletal muscle mass	Upper-body length	Tactile WMA CoP	Knee joint angular data (A ^4^/AV ^5^/AA ^6^)	Tactile sensor width
	Muscle mass	Lower-body length		Ankle joint angular data (A ^4^/AV ^5^/AA ^6^)	Tactile sensor length
	Lean mass	Upper-limb length			
	Body fat mass	Lower-limb length			
		Waist circumference			
		Hip circumference			
		Waist-hip ratio			

^1^ Personal information, ^2^ bioelectrical impedance analysis, ^3^ manual anthropometry, ^4^ angle, ^5^ angular velocity, ^6^ angular acceleration, ^7^ including experimental condition and physical tactile sensor dimensions.

**Table 5:** Comparison of method characteristics with other approaches.

Study	Form Factor	Model	RMSE (mm)		Advantages	Limitations
			ML	AP		
Lee et al. [50]	IMU	ANN	19.5	8.2	A single sensor and a simple structure	Differences between ML and AP
Podobnik et al. [23]	IMU	LSTM	9.0	14.9	Use only statistical models	Model-specific optimal sensor placement
Choi et al. [19]	Insole	LSTM	12.4	37.2	CoP trajectory estimation in an integrated coordinate system	Differences between ML and AP
Duong et al. [18]	FSR Insole + IMU	Bi-LSTM	5.1	14.4	High performance based on multi-sensor	Requires size-matched insoles Differences between ML and AP

## Data Availability

The data presented in this study contain personal information and, therefore, are not publicly available. Requests for data access may be directed to the corresponding author.

## Appendix A. Model Architectures

Figure A1 shows the detailed architectures of CNN Encoder, Residual Block, ResNet Encoder, LSTM Decoder, and Bi-LSTM Decoder that constitute the Bi-LSTM, CNN-LSTM, CNN-Bi-LSTM, and ResNet-Bi-LSTM architectures described in Section 3 of the main manuscript.

## Appendix B. Hyperparameters

Table A1 presents the hyperparameter optimization ranges described in Section 3.4 of the main manuscript. Table A2 provides the final optimized hyperparameter combinations used for training each model, corresponding to the results presented in Section 4 of the main manuscript.

**Table A1:** Hyperparameter search ranges for grid search optimization.

Parameter	Value
Batch Size	[8, 16, 32, 64]
Sequence Length	[11, 15, 19, 23, 25, 27, 29, 31, 33, 35, 37, 39, 41]
RNN Hidden Units	[32, 64, 128, 256]
Intermediate FC Layer Units	[32, 64, 128]
RNN Layers	[2, 3, 4]
RNN dropout	[0, 0.1, 0.2, 0.3]
CNN Output Feature Dimension	[64, 128, 256, 512, 1024]
Weight Optimization Function	Adam, AdamW, RMSProp, SGD, Nadam
Subjects’ Characteristics	gender, height, weight, body fat mass, lean body mass, upper/lower limb length, waist-to-hip ratio
Epochs	10
Learning Rate	0.001
Weight Decay	0.0001
Sliding Window Step	3
Learning Rate Scheduler Step Size	2
Learning Rate Scheduler Decay Factor Gamma	0.5

**Table A2:** Optimized hyperparameter values for each model.

Model	Parameter	Value
Bi-LSTM	Batch Size	16
	Sequence Length	11
	RNN Hidden Units	128
	Intermediate FC Layer Units	32
	RNN Layers	2
	RNN dropout	0.2
	CNN Output Feature Dimension	-
	Weight Optimization Function	RMSProp
	Subjects’ Characteristics	weight, gender, lean mass, upper/lower limb length, waist-hip ratio
CNN-LSTM	Batch Size	8
	Sequence Length	25
	RNN Hidden Units	256
	Intermediate FC Layer Units	32
	RNN Layers	2
	RNN dropout	0.2
	CNN Output Feature Dimension	512
	Weight Optimization Function	RMSProp
	Subjects’ Characteristics	weight, gender, lean mass, upper/lower limb length, waist-hip ratio
CNN-Bi-LSTM	Batch Size	8
	Sequence Length	37
	RNN Hidden Units	128
	Intermediate FC Layer Units	32
	RNN Layers	2
	RNN dropout	0.2
	CNN Output Feature Dimension	512
	Weight Optimization Function	RMSProp
	Subjects’ Characteristics	weight, gender, lean mass, upper/lower limb length, waist-hip ratio
ResNet-Bi-LSTM	Batch Size	8
	Sequence Length	37
	RNN Hidden Units	128
	Intermediate FC Layer Units	32
	RNN Layers	2
	RNN dropout	0.2
	CNN Output Feature Dimension	512
	Weight Optimization Function	RMSProp
	Subjects’ Characteristics	weight, gender, lean mass, upper/lower limb length, waist-hip ratio
